# Research on digital twin diagnosis model for the thermal-electric field of high-voltage switchgears

**DOI:** 10.1038/s41598-025-15626-0

**Published:** 2025-10-01

**Authors:** Feng Ding, Yongji Ma, Xinjue Li, Jiaqi Huang, Jinsong Kang

**Affiliations:** 1 College of Transportation, Key Laboratory of Railway Industry of Maglev Technology, Tongji University, Shanghai, 201804 China; 2 Shanghai Marine Equipment Research Institute, Shanghai, 200030 China; 3 College of Electrical Engineering, Zhejiang University, Hangzhou, 310027 China

**Keywords:** High-voltage switchgear, Digital twin, Finite element simulation, Fault diagnosis, Surrogate model, Engineering, Electrical and electronic engineering, Energy infrastructure

## Abstract

High-voltage switchgear is a critical component in modern power systems, yet it remains vulnerable to insulation degradation and other faults under complex operating conditions. To address these challenges, a digital twin-based online fault diagnosis method is proposed for high-voltage switchgear, integrating thermal and electric field analysis. A three-dimensional model of the KYN28-12(Z) switchgear is first established, incorporating multi-physics simulations to identify key monitoring regions. Building on this, a digital twin surrogate and information model are developed to enable real-time reconstruction and online characterization of coupled thermal-electric fields. For fault feature extraction, optimized classification tree (OCT) and random forest algorithms are employed, while an enhanced adaptive neural-fuzzy inference system (ANFIS) is constructed for intelligent fault diagnosis. Ultimately, the diagnosis model is trained using a combination of finite element simulation data, experimental acquisition data, and on-site operational historical data, ensuring comprehensive learning of switchgear behaviors under various conditions. And the diagnosis relies on data from the digital twin model to achieve accurate virtual-real mapping of switchgear states, providing theoretical support for intelligent operation and maintenance. Experimental results demonstrate a fault recognition rate of 93.4%, with only a 2.3% accuracy drop under 30% noise, verifying the robustness and reliability of the proposed method.

## Introduction

High-voltage switchgear, a critical element in power systems, is vulnerable to insulation aging, partial discharge, and abnormal temperature rise, all of which affect grid reliability. Among all faults in the distribution system, about 37% are caused by the multi-physics coupling effects in switchgear^1^. Traditional offline simulations cannot track real-time state changes^2,3^, and 2D visualization methods fail to capture complex 3D field distributions^4,5^. In recent years, digital twin (DT) technology has become essential for accurately mapping equipment states, diagnosing faults, and enhancing operational efficiency by creating dynamic virtual replicas of physical systems^6^. Therefore, DT-based online fault diagnosis is becoming a promising technology in future power system. research is shifting towards integrating DT technology with multi-source heterogeneous data fusion^7^.

To build accurate power equipment models, a virtual power plant framework integrating grid topology with physical mechanisms has been proposed^8^. Advances in finite element analysis (FEA) have been achieved through semi-empirical calibration methods^9^, and the reliability of dynamic component models has been improved using twin modeling techniques^10^. In interdisciplinary contexts, the integration of industrial internet technologies has enhanced the fidelity of manufacturing system models^11^, while DT validation has demonstrated effectiveness in simulating multi-physics coupled systems, such as solar concentrators^12^. To address real-time interaction constraints, power hardware-in-the-loop technology has been introduced to improve cyber-physical synchronization^13^. However, computational latency remains a critical challenge. Simplified shadow modeling approaches have reduced system complexity at the cost of 3D field resolution^14^, and investigations into nonlinear magnetic material behavior have provided valuable insights into this trade-off^15^. Adaptive meshing strategies have laid the groundwork for dimensionality-reduced surrogate model development^16^.

For dynamic representation, 3D electromagnetic field visualization has been applied to equipment like switchgear^17^, and large-scale parallel computing accelerates simulations^18^. Time-series response optimization^19^ and multi-sensor synchronization^20^ support alarm calibration in complex systems. Despite these advances, traditional numerical methods face efficiency bottlenecks. Transformer-based models improve reconstruction speed^21^, and virtual platforms enhance interaction efficiency^22^. Virtual sensors using adaptive inference reduce localization errors, though single-sensor adaptability is limited by data dimensionality^23^. To further address these challenges, research has shifted toward interdisciplinary data fusion. Multi-parameter fusion aids in complex physical processes^24^, while robust feature extraction advances fault diagnostics^25^. K-nearest neighbors (KNN), valued for its simplicity and spatial adaptability, is widely used for pattern recognition in DT systems. Adaptive neuro-fuzzy inference systems (ANFIS) and optimal classification trees (OCT) offer interpretable, globally optimized fault classification under high feature entanglement. Real-time protocols support fault detection in vertical transport systems^26,27^, and novel sensors such as capacitive-coupling temperature devices extend condition monitoring capabilities^28^. Feature optimization remains critical in complex circuit and microgrid diagnostics^29,30^. Nonetheless, unresolved challenges, such as modeling thermodynamic non-equilibrium in arc plasma^31^ and filtering dynamic interference in mobile communication networks^32^ underscore the need for further breakthroughs. These limitations directly inform the design and innovation of the approach proposed in this study.

In this paper, an online fault diagnosis model for high-voltage switchgear based on DT technology is proposed, integrating real-time sensor data acquisition, 3D field simulation, and intelligent fault diagnosis. The key innovations and contributions are as follows:

1. A high-precision 3D model of the KYN28-12(Z) switchgear is constructed, incorporating a FEA model for coupled thermal-electric fields. Experimental data are leveraged to precisely calibrate insulation and temperature-rise risk zones, laying a foundation for high-fidelity DT modeling.
2. A reduced-order surrogate model is developed through the integration of mesh coarsening, dictionary tree deduplication, and KNN algorithms, enabling efficient and continuous DT simulations across diverse operating conditions. To enhance dynamic visualization of multi-physics fields, a local-loop communication protocol and 3D animation-based interactive system are designed.
3. An ANFIS-OCT hybrid diagnostic framework is proposed, which combines the interpretability of OCT with the adaptive learning capability of enhanced ANFIS, significantly improving fault recognition accuracy under complex operating conditions.

### Fault diagnosis system structure based on digital twin

**Fig. 1:**
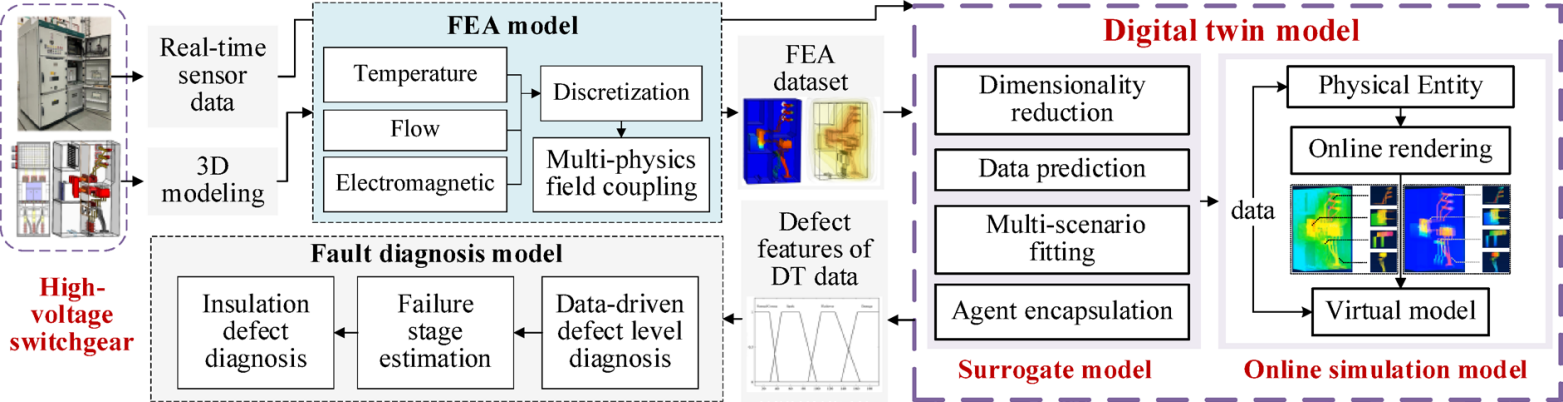
Fault diagnosis process of high-voltage switchgear based on digital twin model.

The research framework is illustrated in Fig. 1. FEA model relies on offline computation to model physical fields, making it difficult to monitor equipment operation in real time. DT technology addresses this limitation by leveraging sensor data and historical information to construct a virtual replica of the physical entity, enabling online simulation. This approach allows for intuitive interpretation of real-time sensing data and synchronous simulation of equipment states without the need for high-performance cloud servers or high-bandwidth networks, thereby enhancing situational awareness and responsiveness.

The data underpinning the digital twin model is derived from a combination of finite element multi-physics simulation data, experimental measurements, and extensive historical records. This study integrates FEA model with experimental data to construct a multi-condition simulation dataset. A dimensionality reduction algorithm is employed to lightweight the 3D point cloud of the switchgear. Based on this, predictive algorithms are used to develop a surrogate model of DT nodes under various working conditions. The system outputs twin information of the switchgear in response to real-time sensor data, supporting online simulation and fault diagnosis. This enables real-time evaluation of equipment status and defect identification in a virtual environment.

### Finite element simulation coupling of thermal-flow-electric fields

**Fig. 2:**
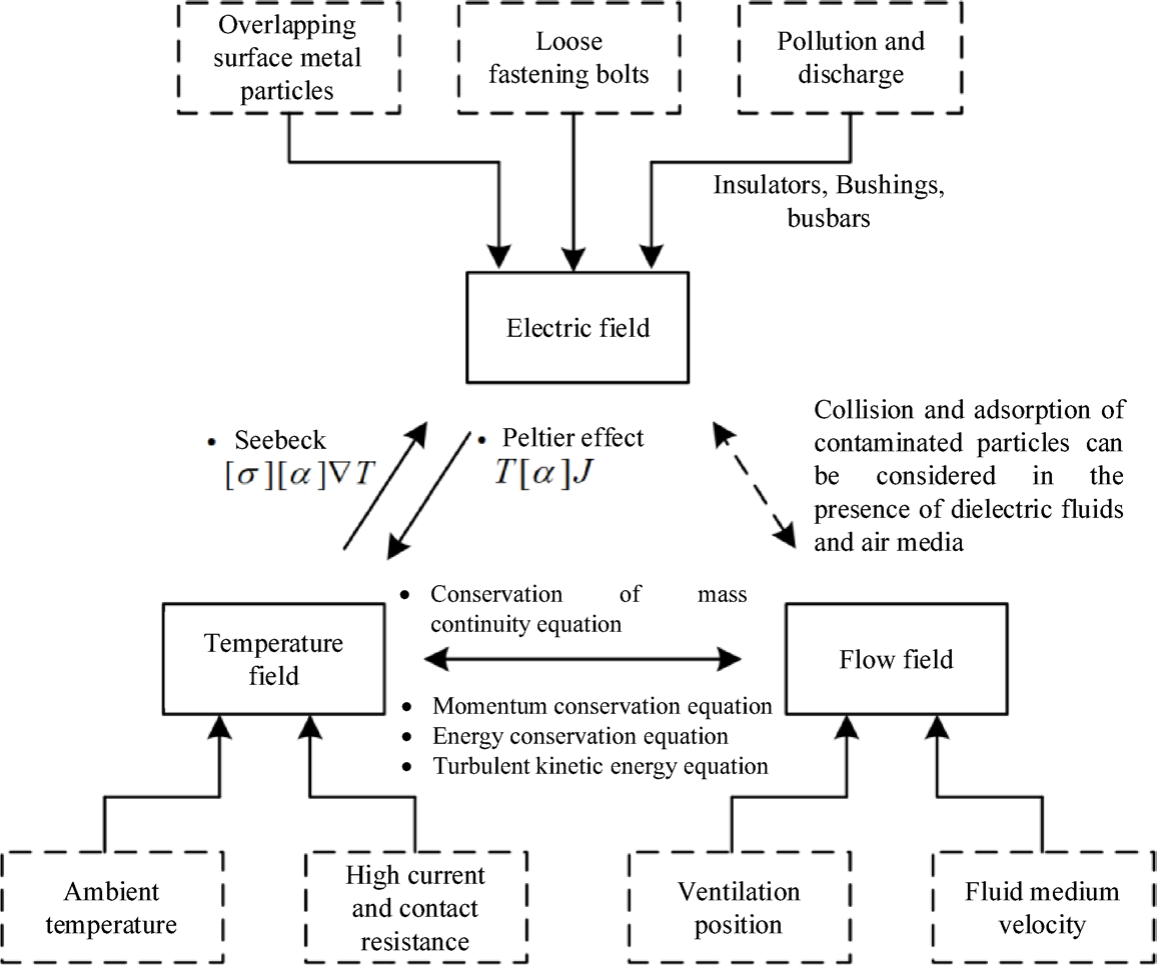
Multi-physics coupling relationships.

Additionally, in high-voltage switchgear, the electric field distribution is affected by structural assembly, the presence of metallic particles, and loosened fastening screws, and the thermal field is influenced by ambient temperature, operating current, and contact resistance. The interplay between the thermal and electric fields is driven by the Peltier and Seebeck effects, forming a coupled system. For the KYN28-12 switchgear, which primarily relies on natural convection for heat dissipation, the internal flow field is directly coupled with the thermal field through the conservation of mass, momentum, and energy equations, as shown in Fig. 2. Furthermore, the interaction between contamination particles and the electric field introduces additional indirect coupling effects. This complex multi-physics interaction needs an integrated approach to modeling and simulation, enabling a comprehensive DT representation of the switchgear’s thermal-electric-flow behavior.

A three-dimensional model of the KYN28-12(Z) high-voltage switchgear and performs multi-physics coupling thermal-electric field simulations is constructed. Based on the FEA data, a DT surrogate model is developed, and a complete DT model of the switchgear is further constructed to enable real-time simulation and analysis of the internal coupled electric-thermal-fluid fields.

### Dimensionality reduction of 3D nodes and construction of reduced-dimensionality dataset

**Fig. 3:**
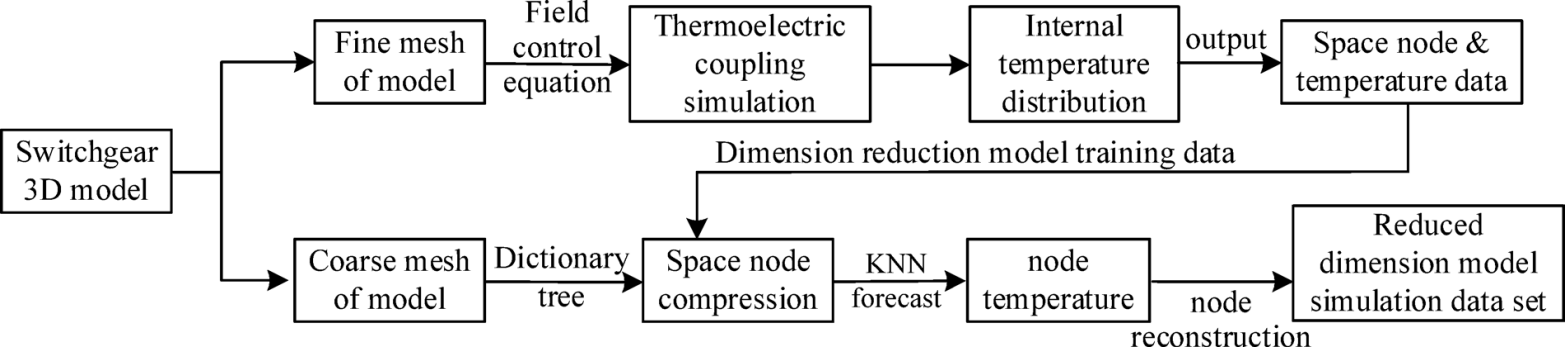
Dimensionality reduction model reconstruction workflow.

High-voltage switchgear contains numerous internal components with complex structures, resulting in a significant increase in problem dimensionality when constructing surrogate models. This not only increases the number of sample points and computational time but also reduces model accuracy and weakens the reliability of extracted information. To overcome this problem, this study proposes a mesh coarsening and dictionary tree deduplication algorithm to effectively compress switchgear node data, further construct a dimensionality reduction dataset for surrogate models to efficiently approximate the behavior of complex systems. as shown in Fig. 3. While mesh coarsening and dictionary trees are mature technologies, their integrated implementation offers unique advantages for switchgear digital twin models. In terms of balancing accuracy and efficiency, a 90% node reduction is achieved while maintaining field extreme value errors below 5%. Regarding physical consistency, coarsening preserves topological dependencies, while deduplication maintains field continuity. Regarding real-time feasibility, the optimization of spatial nodes by both methods significantly reduces proxy model response time, far exceeding the speed of traditional finite element analysis. This optimized workflow addresses the critical gap between high-resolution simulation and real-time constraints in digital twin models of power equipment.

The mesh coarsening combined with KNN algorithm for finite element fine mesh simulation model reconstructs the reduced dimensional spatial nodes and extracts thermal and electric field features; After traversing the coarse grid, the dictionary tree maps all nodes into a three-layer tree structure, eliminating redundant nodes by covering duplicate data and reducing unnecessary character comparisons.

As a lazy learning method and non-parametric model, the KNN algorithm efficiently processes large-scale simulation datasets while demonstrating strong robustness against outliers.

Taking the temperature field as an example, assume that the fine-mesh simulation dataset contains all node positions and their corresponding temperatures, while the dimensionality-reduced model *x* nodes serve as test samples, the Euclidean distance between ${x_i}$ and the dataset *X* is given by:

1 $$d({X_j},{x_i})=\sqrt {{{({X_j}_{x} - {x_i}_{x})}^2}+{{({X_j}_{y} - {x_i}_{y})}^2}+{{({X_j}_{z} - {x_i}_{z})}^2}}$$

where the K nearest neighbors of ${x_i}$ are denoted as ${X_i}_{k}=\{ {X_{i+1}},{X_{i+2}}, \cdot \cdot \cdot ,{X_{i+k}}\}$. Using the K-nearest neighbor rule, the temperature attribute of node ${x_i}$is estimated by the mean or weighted mean of its nearest neighbors’ temperature values ${X_{ik}}$. When inverse distance weighting ${\omega _j}$ is applied, the weighted output is computed as:

2 $$\begin{aligned} \left\{ \begin{gathered} {x_i} = (\mathop \sum \limits_{j = 1}^k {\omega _j} \cdot {X_{i + j}})/(\mathop \sum \limits_{j = 1}^k {\omega _j}) \\ {\omega _j} = 1/d({X_j},{x_i}) \\ \end{gathered} \right. \end{aligned}$$

Assuming that the temperatures of the KNN follow a random variable distribution ${T_i}$, the posterior probability $P({T_i}|{X_{ik}})$ is defined based on the law of large numbers. If the dataset is sufficiently large, the proximity ${X_i}_{k}$ of ${x_i}$ to its nearest neighbors ensures $P({T_i}|{X_{ik}}) \approx P({T_i}|{x_i})$. Thus, the rule provides an effective approximation for estimating the temperature attribute ${T_i}$ of node ${x_i}$.

### Implementation of the surrogate model under abnormal conditions in switchgear

The core objective of the surrogate model is to replicate the behavior of the original system using a simplified mathematical representation, thereby reducing computational resource demands and enabling real-time DT synchronization. This study employs the radial basis function (RBF) interpolation method, which approximates complex three-dimensional point cloud data through a weighted sum of basic functions. RBF-based surrogate models exhibit strong robustness and adaptability in nonlinear data fitting while imposing no specific constraints on response characteristics. Therefore, based on the dimensionality-reduced simulation dataset, we construct a DT surrogate model using the RBF interpolation algorithm.

The construction process consists of the following steps: (1) Using the KNN algorithm, we process finite element fault simulation and ambient temperature simulation datasets, obtaining six sets of reduced-order simulation data related to insulator contamination faults and six sets related to ambient temperature variations. (2) To enhance computational efficiency, we construct RBF interpolation functions for 29,400 reduced-order nodes. (3) Assuming each spatial node is represented as (*x*_*i*_,*y*_*i*_,*z*_*i*_), and each node has *N* simulation values (*T*_*i*_,*t*_*i*_), where *i = 1*,*2*,*…*,*N*, the RBF interpolation function can be expressed as:

3 $$\hat {f}(T)=\sum\limits_{{i=1}}^{N} {{\omega _i}\varphi (||T - {T_i}||)}$$

where, *N* denotes the number of simulation value sets. The relationship between each data point and the interpolation center is determined using a Gaussian kernel function:

4 $$\varphi (T)=\exp \left( { - \frac{{||T - {T_i}|{|^2}}}{{2{\sigma ^2}}}} \right)$$

where, *T* represents temperature data points, *T*_i_ denotes interpolation centers, and *σ* is the standard deviation controlling the kernel function width. Using the radial basis function, we construct an interpolation matrix $\Phi= [\varphi_{ij}]$ , leading to the system of equations:

5 $$\left[ {\begin{array}{*{20}{c}} {{\varphi _{11}}}&{{\varphi _{12}}}& \cdots &{{\varphi _{1N}}} \\ {{\varphi _{21}}}&{{\varphi _{22}}}& \cdots &{{\varphi _{2N}}} \\ \vdots & \vdots &{}& \vdots \\ {{\varphi _{N1}}}&{{\varphi _{N2}}}& \cdots &{{\varphi _{NN}}} \end{array}} \right]\left[ {\begin{array}{*{20}{c}} {{\omega _1}} \\ {{\omega _2}} \\ \vdots \\ {{\omega _N}} \end{array}} \right]=\left[ {\begin{array}{*{20}{c}} {{t_1}} \\ {{t_2}} \\ \vdots \\ {{t_N}} \end{array}} \right]$$

where $W=[{\omega _i}]$ is the coefficient matrix and with ${\varphi _{ij}}={\varphi _{ji}}=\varphi (||{T_j} - {T_i}||)$, so the *j*-th row in the matrix expression can be expressed.

6 $$\hat {f}({T_j})={t_j}=\sum\limits_{{i=1}}^{N} {{\omega _i}\varphi (||{T_j} - {T_i}||)}$$

To minimize errors, the optimal coefficients are estimated using the least squares method:

7 $$W={({\Phi ^T}\Phi )^{ - 1}}{\Phi ^T}t={\Phi ^{ - 1}}t$$

The interpolation functions for nodes across six different ambient temperature conditions form the ambient temperature surrogate model. Similarly, six sets of fault-induced temperature rise data $\hat {f}(\Delta t)=\sum\nolimits_{{i=1}}^{N} {{\varpi _i}{\varphi _t}(||t - {t_i}||)}$ are used to construct the fault temperature rise surrogate model, and then the final system temperature can be obtained as follows:

8 $$T(x,y,z)={[\hat {f}(T)+\hat {f}(\Delta t)]_{(x,y,z)}}$$

To facilitate seamless storage, retrieval, and deployment of the surrogate model, the Python’s Pickle module is used to store the trained model as a PKL (Pickle) file. The pickling process efficiently serializes complex Python object structures into byte streams for storage, while the unpickling process reconstructs the original objects, and thus the efficient data sharing and model deployment across different Python programs can be achieved.

In this study, an evaluative method based on trustworthiness and continuity indicators is used to analysis the dimensionality reduction results^33^. Trustworthiness quantifies the similarity between the surrogate model and finite element simulation, as shown in Eq. (9). Continuity characterizes the preservation of local relationships in the reduced space relative to the original space, as expressed in Eq. (10).

9 $$M_{1}^{{(K)}}=1 - \frac{{2\sum\limits_{{i=1}}^{N} {\sum\limits_{{j \in {U_k}(i)}} {[d({X_j},{x_i}) - K]} } }}{{NK(2N - 3K - 1)}}$$

10 $$M_{2}^{{(K)}}=1 - \frac{{2\sum\limits_{{i=1}}^{N} {\sum\limits_{{j \in {V_k}(i)}} {[d({X_j},{x_i}) - K]} } }}{{NK(2N - 3K - 1)}}$$

where, ${C_k}({x_i})$ and ${\hat {C}_k}({x_i})$ represent the nearest neighbor sets in the original and dimensionality-reduced spaces, respectively. Based on this, set ${U_k}(i)$ is defined as representing ${x_i} \in {\hat {C}_k}({x_i}) \wedge {x_i} \notin {C_k}({x_i})$, while set ${V_k}(i)$ represents ${x_i} \notin {\hat {C}_k}({x_i}) \wedge {x_i} \in {C_k}({x_i})$.

### Digital twin online simulation based on information model

To enable real-time output of the developed DT surrogate model and accurately reflect the thermal-electric field distribution of the switchgear, this study establishes an online DT simulation based on an information model.

The typical defects and faults in high-voltage switchgear can be categorized as follows: (a) Abnormal internal heating: temperature rise due to contact resistance at cables, terminals, and connection points. Eddy currents and hysteresis losses in conductors and transformers under varying magnetic fields. Abnormal temperature increases due to aging, contamination, or damage to internal components and insulation materials. (b) Contaminated post insulator discharge. (c) Partial discharge faults in cable joint insulation defects. To monitor the condition of high-voltage switchgear, a multi-sensor condition monitoring system is designed. This system consists of three core components: Information Acquisition: Sensors collect thermal and electrical field data in real time. Data Communication: Secure and efficient transmission of collected sensor data. Condition Monitoring: analyzing and visualizing real-time system status.

### Digital twin data information model and communication scheme

The DT system creates a high-precision virtual model to simulate and analyze the operating status of switchgear in real time. To ensure efficient and reliable data transmission, a Socket-based TCP communication protocol is implemented for local loopback transmission. The thermal-electric field surrogate models are serialized as PKL files, which enable efficient storage and retrieval. Additionally, Python’s Pandas and NumPy libraries are utilized to process and analyze both structured and unstructured data. The real-time sensor monitoring data undergo preprocessing and feature extraction in Python before being transmitted via local loopback communication to Unity 3D animation software, where the DT visualization is rendered.

The finite element simulation outputs mesh node and point cloud data in STL triangular facet format, enabling data reconstruction. To replicate spatial nodes and visualize the thermo-electric field in Unity, a detailed analysis of triangular mesh structures is essential. In a 3D Cartesian coordinate system, triangle vertices follow the right-hand rule based on surface normal. For instance, triangles A (a, b, c) and B (b, c, d) share vertices b and c, resulting in duplicate data. To eliminate redundancy and optimize rendering, a precomputed vertex index structure is introduced. Each triangle initially includes six vertices, but shared vertices are deduplicated, leaving only unique vertices (e.g., a, b, c, d). Each is assigned an index (1, 2, 3, 4), and a mapping function links original mesh vertex to these indices. This indexed sequence efficiently reconstructs the mesh for real-time DT simulation. To implement this in Unity, a Mesh defines its geometry using a Vector3 array and triangle indices, with UV coordinates enabling texture mapping. Visual effects are controlled by assigning a Material and Shader. The Mesh is attached to a GameObject and rendered via Mesh Filter and Mesh Renderer components. Electric and temperature field data are received through local Python Socket communication, nonlinearly mapped to HSV color space, and applied to the Mesh material.

### Fault diagnosis and assessment based on digital twin architecture

DT surrogate model is relied to accurately output data reflecting the thermal-electrical field distribution characteristics. Real-time data output is achieved through an information model, providing credible data support for subsequent state assessment and fault diagnosis. Ultimately, by combining field data with DT surrogate model data, a training set and test set are formed for the development of the fault diagnosis model of the high-voltage switchgear. Under the condition of a small training set, OCT can effectively identify irrelevant features and reduce their importance to zero. Its global optimization characteristics ensure that irrelevant features are eliminated with minimal cost. Therefore, a combination of OCT and random forest algorithms is chosen for feature importance prediction. The process primarily includes:

#### (1) decision tree objective function and split criteria construction

11 $$\left\{ \begin{gathered} {\min _{Tree}}\left( {Loss(Tree) + \lambda \cdot C(Tree)} \right) \hfill \\ IG(S,A) = E(S) - \sum\nolimits_{v \in Values(A)} {\frac{{|{S_v}|}}{{|S|}}E({S_v})} \hfill \\ \end{gathered} \right.$$

where min_*Tree*_ denotes the minimization loss function. *IG*(*S*,*A*) represents the information gain, *Loss*(*Tree*) is the prediction error, and λ is the regularization parameter. *C*(*Tree*) is the complexity of the tree. *S* is the current node’s data set, *A* is the feature, *S*_*v*_ is the subset after splitting, and *E(S)* represents the entropy of the dataset.

#### (2) feature importance evaluation

12 $$FI(A) = \sum\nolimits_{t \in A} {{\Delta}\,Loss(t)}$$

Feature importance *FI*(*A*) is quantified by calculating the reduction in prediction error due to the feature A at the split point, where Δ*Loss*(*t*) represents the loss reduction at node *t* when feature A is used.

#### (3) optimization and solution

Mixed integer optimization (MIO) is used to solve the optimization problem, involving the construction of integer linear or nonlinear formulations to represent decision trees and feature evaluation, with an optimization solver providing the optimal solution.

### Fault diagnosis model construction and training process for switchgear

The ANFIS inference model consists of two types: the Mamdani model and the Takagi-Sugeno model. The Mamdani model processes the output as a fuzzy set variable, while the model outputs either a zero-order constant value or a linear combination of input variables. Both models have an inference structure with five layers: input, membership, rule, decision, and output. Compared to the Mamdani model, the model output, typically zero-order constant values or first-order linear combinations, is more conducive to the adaptive parameter adjustment process and output analysis. For the model, assuming the input vector is xx, the fuzzy linguistic expression for the input components is:

13 $$T({x_i})=\{ A_{i}^{1},A_{i}^{2}, \cdots ,A_{i}^{{{m_i}}}\} |x={[{x_1},{x_2}, \cdots ,{x_n}]^T}{\text{ }}(i=1,2, \cdots ,n)$$

where $A_{i}^{j}(j=1,2, \cdots ,{m_i})$ is the *j*-th linguistic variable value of input *x*_*i*_ and affiliation.

14 $${u_{A_{i}^{j}}}({x_i}) \, \& \, (i=1,2, \cdots ,n;j=1,2, \cdots ,{m_i})$$

The fuzzy rule condition is denoted by ${x_1}{\text{ is }}A_{1}^{j}{\text{ and }}{x_2}{\text{ is }}A_{2}^{j}{\text{ and}} \cdots {\text{and }}{x_n}{\text{ is }}A_{n}^{j}$, where the weight of each fuzzy component through different membership functions is represented by ${p_{jl}}$, and the output of each rule is given by:

15 $${y_j}={p_{j0}}+{p_{j1}}{x_1}+ \cdots +{p_{jn}}{x_n}$$

The fitness of each rule is represented by *a*_*j*_, and the system’s output variable y is expressed as a weighted average of the outputs from each rule:

16 $$y={{\sum\nolimits_{{j=1}}^{m} {{a_j}{y_j}} } \mathord{\left/ {\vphantom {{\sum\nolimits_{{j=1}}\phantom{0}^{m} {{a_j}{y_j}} } {\sum\nolimits_{{j=1}}\phantom{0}^{m} {{a_j}} }}} \right. \kern-0pt} {\sum\nolimits_{{j=1}}\phantom{0}^{m} {{a_j}} }}{\text{ | }}{a_j}={u_{A_{1}\phantom{0}\phantom{0}^{j}}}({x_1}){u_{A_{2}^{j}}}({x_2}){\cdots}{u_{A_{n}\phantom{0}^{j}}}({x_n})$$

In this research, an adaptive fuzzy neural network based on the Takagi-Sugeno inference model is constructed. The model establishes a nonlinear mapping relationship between input and output variables, and in the iterative process, the input-output data pair is modeled through error backpropagation. The optimal weight of the fuzzy membership function is calculated through local approximation during data flow, ensuring fast computational speed, adaptive learning, and fuzzy inference capabilities.

The ANFIS architecture employs a hybrid learning mechanism to mitigate error propagation across layers. In the forward pass, least-squares optimization adjusts consequent parameters to minimize output errors. In the backward pass, gradient descent updates antecedent parameters of membership functions. This two-stage optimization decouples error propagation paths, preventing cumulative amplification. Let $\Upsilon$ (set to 0.15 via empirical tuning) controls error sensitivity, and *η* represents inherent noise tolerance.

Specifically, the error term *δ*_*k*_ for layer *k* is bounded by:

17 $$|{\delta _k}| \leqslant \Upsilon \cdot \hbox{max} ({\delta _{k - 1}})+\eta$$

The layers I, II, III, IV, and V represent the input layer, membership layer, rule layer, decision layer, and output layer, respectively. The frontend network performs the fuzzification, rule formation, and decision-making functions of the input variables, while the backend network calculates the weights of input variables for each rule, ultimately generating the system output.

**Fig. 4:**
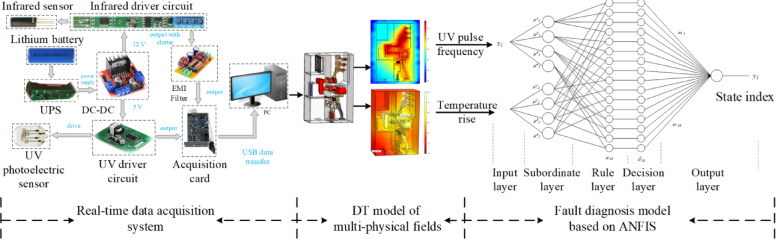
Fault diagnosis system network structure.

In this study, the field data combined with the DT surrogate model data form the training and testing data sets for the high-voltage switchgear fault diagnosis model. Based on this, the fault diagnosis model is constructed, and the training and diagnostic verification processes are completed. In the defect environment of the high-voltage switchgear, external voltage differences may result in corona discharge, spark discharge, or even sustained electric arcs, leading to flashover breakdown of the equipment, rapid temperature rise, intense discharges, and total destruction of insulation. Therefore, sensor monitoring data is transmitted to a PC via a state-sensing system. To ensure real-time information collection, an MLX90614 infrared sensor (0.5 °C accuracy) and an R13192 UV photoelectric sensor (spectrum 185–260 nm) are used in conjunction with an NI-USB6210 data acquisition card. This allows for a 10 K acquisition frequency of thermal and electrical information, which is transmitted to the PC in real time. Subsequently, a temperature and pulse data processing algorithm on the PC extracts the maximum temperature rise per unit time and the UV pulse frequency as input variables *x*_1_ and *x*_2_ for the Takagi-Sugeno model. To achieve broader domain coverage, four trapezoidal membership functions are defined for each of the two input variables, resulting in 16 fuzzy inference rules and normalized outputs at the decision layer. The state index y_1_ is subsequently derived according to Eq. (16). The architecture of the diagnostic system network is illustrated in Fig. 4. The two types of data used in this study are sourced from finite element multi-physics simulation data, experimental measurements, and historical operational records. The integration of these diverse datasets enhances the robustness and reliability of the diagnostic model by capturing both the physical behavior under controlled conditions and real-world operational variations.

**Fig. 5:**
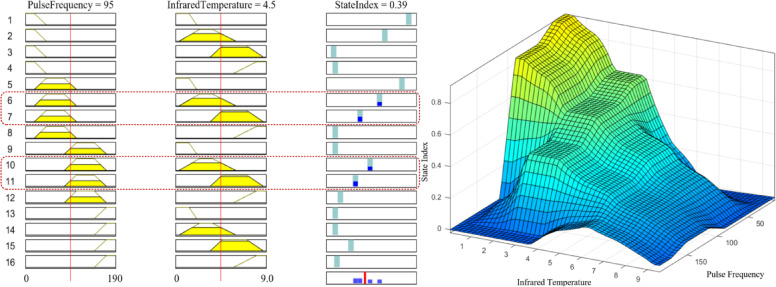
ANFIS training results: (**a**) state index output process; (**b**) input variable-output index relationship.

The network training results are shown in Fig. 5. Figure 5(a) demonstrates the state index output process through the membership function, where the input pulse frequency *x*_1_ and infrared temperature rise *x*_2_ are included in the membership function domain, and the weighted sum of the corresponding rule outputs represents the system output, the state index *y*_1_. Figure 5(b) visualizes the nonlinear mapping relationship between the input variables and the output variable in three-dimensional space. When definite *x* and *y* inputs exist, the corresponding output *z*-axis state index can be obtained.

## Results

### Construction of the switchgear field simulation dataset

The switchgear field simulation dataset forms the foundation for constructing the DT surrogate model. This research primarily focuses on the finite element simulation model construction and the process of obtaining the thermal-electrical field simulation dataset.

#### (1) finite element simulation model construction

**Fig. 6:**
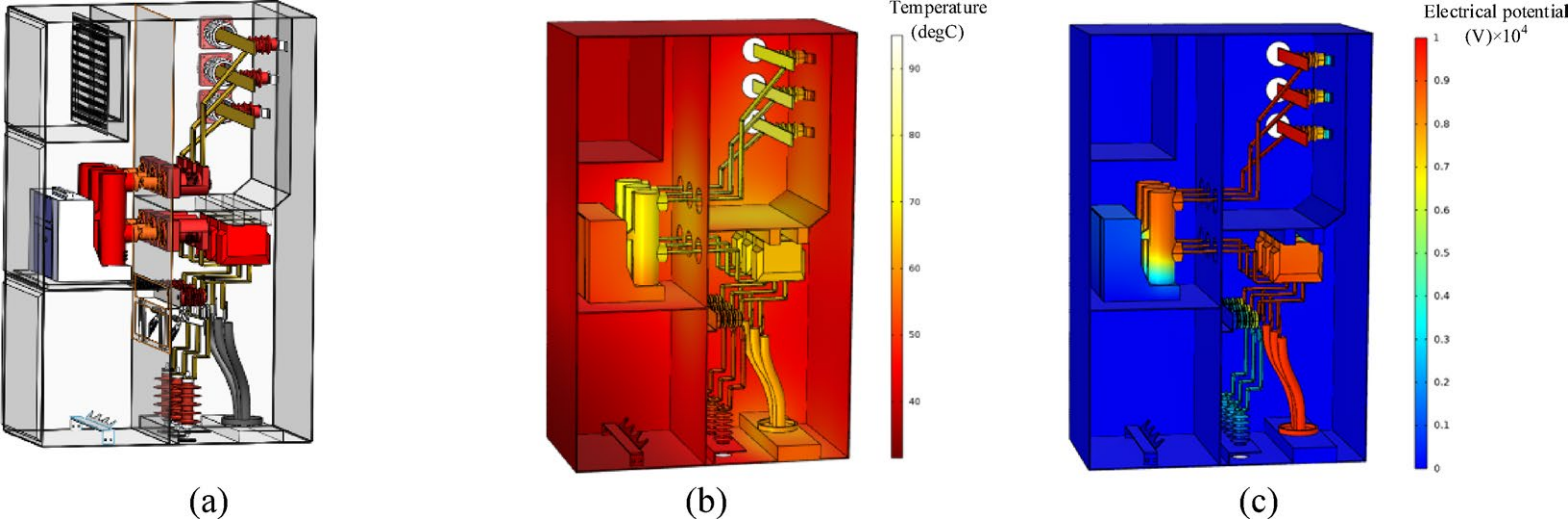
Simulation results under normal operating conditions: (**a**) 3D diagram (**b**) thermal-flow field (**c**) electrical field.

In accordance with the physical materials of the switchgear and the standard for the artificial contamination test of high-voltage insulators in AC systems, which specifies the reference conductivity of the contamination layer, the construction of the dataset involves material parameters as shown in Table 1. The three-dimensional model of the switchgear and the thermal and electrical field results under rated conditions and ambient temperature are illustrated in Fig. 6.

**Table 1:** Material electrical parameters.

Material	Copper	Epoxy resin	Air	Wet contamination	Dry contamination
Relative permittivity	1 × 10^13^	3.9	1.0006	10	3
Conductivity (S/m)	5.99 × 10^7^	1 × 10^−15^	1 × 10^−14^	10	1 × 10^−14^
Specific heat (J/(kg·K))	385	19	1005	628	860
Thermal conductivity (W/(m·K))	400	0.2	0.025	0.6	0.4

#### (2) Thermal-flow simulation dataset acquisition

Due to the strong coupling between temperature and flow fields, both device defects and ambient temperature affect the thermal distribution within the switchgear. To capture more distinct temperature variation patterns, simulations were conducted on the temperature rise of the three-phase post insulators on the grounding switch under polluted conditions. For dry pollution scenarios, fine-mesh finite element simulations were performed across ambient temperatures ranging from 10 °C to 35 °C, yielding results consistent with the rated conditions of the KYN28 switchgear. As ambient temperature increases, the overall thermal pattern remains stable, with the maximum temperature rise (56.7 ~ 57.7 °C) consistently occurring at the bend of phase B busbar and a phase difference of less than 1 °C. The lowest temperatures (11.1 ~ 12.2 °C) appear on the outer shell. Higher ambient temperatures reduce heat dissipation, slightly increasing the temperature rise, which aligns with physical laws and supports use as a simulation dataset for varying conditions. Under wet pollution coverage, the maximum temperature on the insulator surface reaches 60.7 °C, with fault currents causing an approximate 4.3 °C rise in the busbar and circuit breaker inlet. All simulated conditions conform to operational and fault characteristics, providing multiple datasets (0%~100% pollution moisture levels) for surrogate model training.

#### (3) current field simulation dataset acquisition

In order to enhance the electric field simulation data set, considering that defects such as contamination on the support insulators inside the switchgear will cause uneven distribution of the electric field, and the national benchmark measurement range of the conductivity of the contaminated electrolyte solution in the contamination test of high-voltage equipment is 5 ~ 20 S/m when the measurement uncertainty is 0.05%~0.07%, especially for the artificial contamination test of high-voltage insulators in the AC system, the contamination conductivity values of different contamination levels range from 5 ~ 10 S/m, and dry contamination has almost no conductivity. Therefore, the contamination test of the insulation defects of the support insulators and cable terminals was focused on for feature analysis and fault modeling. By changing the surface attachment material of the insulator in the simulation model, the fault simulation was achieved. This paper simulated the working conditions of the A-phase grounding switch insulator surface covered with different degrees of wet contamination: when the support insulator was covered with dry contamination, the insulation was good at this time, and the electric field on the surface of the A-phase insulator dropped evenly; when there was 50% wet area contamination coverage, the electric field on the surface of the Phase A insulator is distorted. The wet zone (0 ~ 0.12 m) experiences a 2 kV potential drop, while the dry zone (0.12 ~ 0.26 m) experiences an 8 kV voltage difference. However, the insulator maintains its insulation performance, and the field distribution in the rest of the switchgear is virtually unaffected. When the insulator is completely covered by contamination, the distortion of the Phase A insulator’s electric field increases, and the entire insulator (0 ~ 0.26 m) can only withstand a 2.5 kV voltage drop. This also distorts the electric field in the grounding switch’s steel support structure and the copper busbar. Therefore, considering that the insulator maintains insulation performance before reaching 50% wetness, this paper adds further simulations for operating conditions with 25%, 65%, and 80% wetness. Data sets are generated based on the coverage (0–100%) and location of the wet zone for different contamination levels to supplement the alternative model.

### Dimensionality reduction of 3D point cloud data for switchgear DT surrogate model

**Fig. 7:**
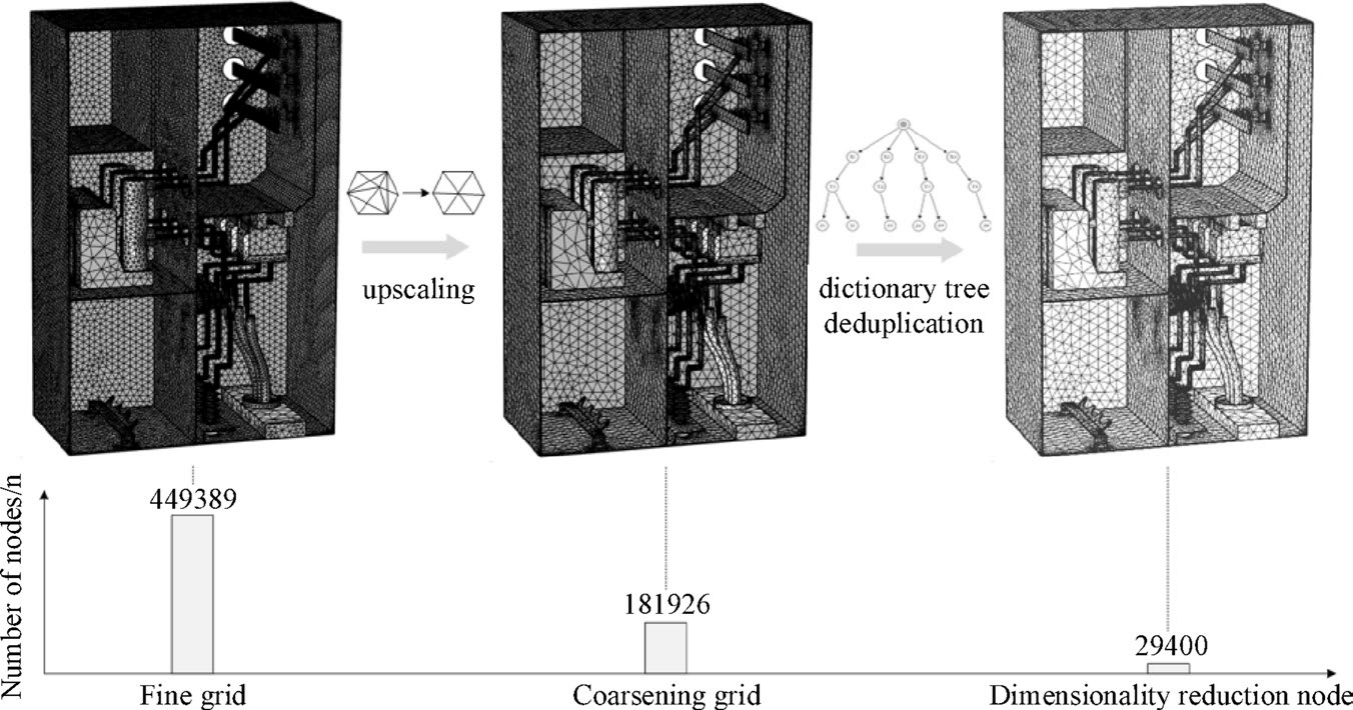
Dimensionality reduction of switchgear spatial nodes.

As shown in Fig. 7, based on the fine mesh of the finite element model, various switchgear components were appropriately coarsened. Structural components with minimal impact on simulation results were assigned a coarser mesh. This reduced the number of nodes by 59.5%, from 449,389 to 181,926. The spatial nodes in the simulation model were based on the STL format in a three-dimensional Cartesian coordinate system, where triangular meshes were formed by vertex points and the right-hand rule. However, redundant and shared nodes remained. To further reduce dimensionality, a dictionary tree method was applied, decreasing the number of nodes to just 6.5% of the original finite element simulation model’s nodes.

### Analysis of the switchgear digital twin surrogate model

**Fig. 8:**
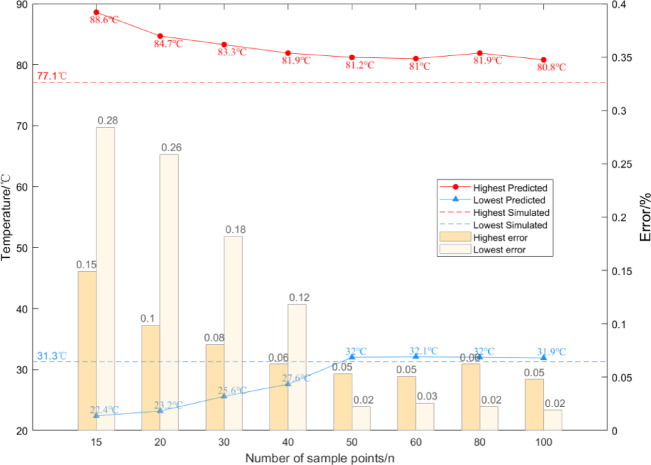
Temperature extremes at different K Values.

To validate the reduced model nodes, simulation data of the switchgear at an ambient temperature of 20 °C were used, with the KNN algorithm applied at *K* values ranging from 15 to 100. The reconstructed cloud map, generated by predicting the nodes of the reduced model (corresponding to (x, y, z, T) in COMSOL), is shown in Fig. 8. By comparing these results with the finite element simulation model, it is clear that for small *K* values, the model’s predictions are heavily influenced by a few nearest neighbors, leading to an uneven cloud map and significant deviations in predicted values.

When *K* ≤ 50, the temperature cloud map shows a clear upward shift in overall temperature, indicating a rise in the average temperature within the switchgear. The predicted maximum and minimum temperatures deviate from finite element results by 14.9% and 28.4%, respectively, with noticeable discrepancies in the locations of temperature extremes. As *K* increases, the temperature distribution shifts downward, and prediction errors for extreme temperatures gradually decrease. At *K* ≥ 50, the temperature map becomes more uniform and closely aligns with the finite element simulation, with errors in maximum and minimum temperatures reduced to below 5% and 2%, respectively.

Considering the trade-off between model accuracy and computational complexity, *K* = 50 was selected for reduced-order modeling. A mean-weighted KNN algorithm was applied to construct the reduced model using 50 sample points. The resulting temperature node model demonstrated strong agreement with finite element simulations, validating its reliability and accuracy. With an ambient temperature of 17 °C and an insulator wet-zone fault index of 50%, the reconstructed cloud map yielded a 2.3% overall error. The maximum temperature of 81.5 °C at the B-phase busbar corner showed a 4% deviation, while the minimum temperature of 29.7 °C on the switchgear surface had only a 1.3% error. Evaluation metrics of model consistency, *M*_1_ = 0.9273 and *M*_2_ = 0.9362, further confirm the model’s capability to accurately capture the temperature field.

**Fig. 9:**
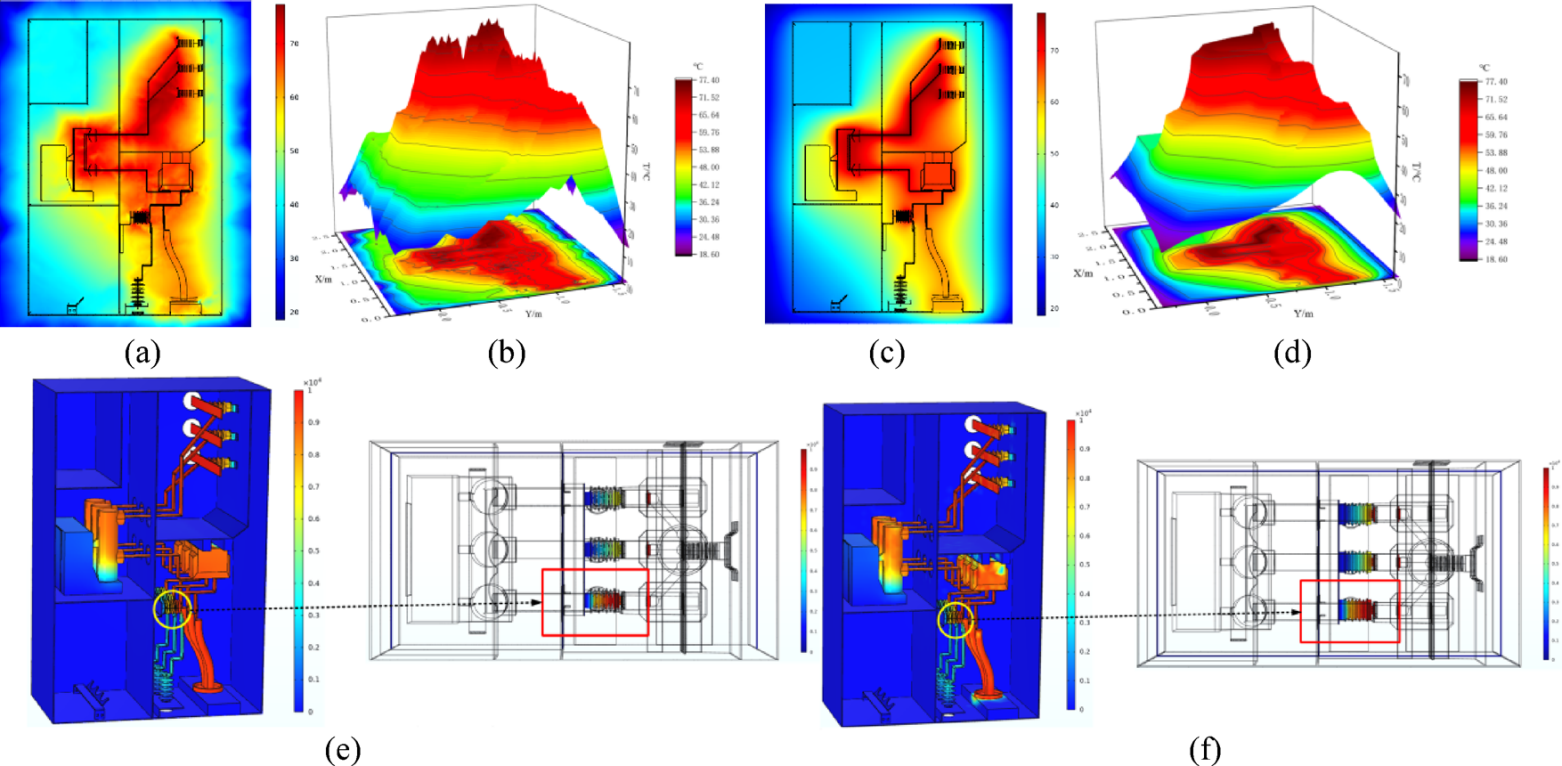
Comparison of temperature surrogate model output: (**a**) surrogate model cloud map cross-section; (**b**) cross-section terrain map; (**c**) finite element simulation cross-section; (**d**) cross-section terrain map; (**e**) Electric field finite element simulation; (**f**) Electric field proxy model output.

In terms of computational efficiency, the PKL-based temperature rising surrogate model achieved rapid inference: the ambient and fault-temperature models required only 3.63 s and 3.12 s, respectively, during deserialization. Subsequent outputs of temperature node data took 0.19 s and 0.13 s, resulting in a total response time of 0.32 s for the entire model, supporting real-time applications. As shown in Fig. 9, temperature cross-section comparisons reveal that although some patterned artifacts appear in planar regions due to the dataset-driven KNN approach, the overall temperature field remains consistent with finite element results. The reconstructed distribution effectively captures key thermal features of the switchgear, particularly in high-temperature concentration zones.

In addition to its application in the thermal field, the KNN-based anomaly processing method is also applicable to the electric field data, with consideration given to the coupling mechanisms between multiple physical domains.

Additionally, the terrain contour map corresponding to the cross-section was plotted. The reconstructed terrain map showed a high degree of agreement with the finite element simulation’s terrain map, with slight spikes in high-temperature regions due to the nature of the algorithm. However, the consistency in the regions of maximum and minimum temperatures is high, which further validates the reliability and effectiveness of the surrogate model.

To improve visualization speed and reduce computer resource demand, the same 29,400 reduced nodes were used. Based on the KNN data prediction algorithm, a reduced electrical field simulation dataset was constructed. Simultaneously, the RBF surrogate model algorithm was employed to calculate the radial basis function for the 29,400 nodes across six simulation datasets. The system’s DT electrical field surrogate model was generated via a PKL file. When the insulator wet zone was set to 50%, the surrogate model’s node electrical field data showed some distortion around the transformer and metal bracket contact areas, but most regions closely matched the finite element simulation. The model’s continuity and reliability, with *M*_1_ = 0.9141 and *M*_2_ = 0.9197, validated the reliability of the electrical field surrogate model.

In fact, the ambient temperature range considered in this study spans from 10 °C to 40 °C, while the insulator wet-zone conditions cover the full interval from 0 to 100% humidity. Corresponding simulation datasets were generated across these ranges to reflect typical operational scenarios, capture variations in insulation performance under different humidity levels, and account for the strong coupling between temperature and flow fields within the switchgear, forming the basis for subsequent surrogate model development.

### Digital twin-based online simulation and effectiveness verification of switchgear

**Fig. 10:**
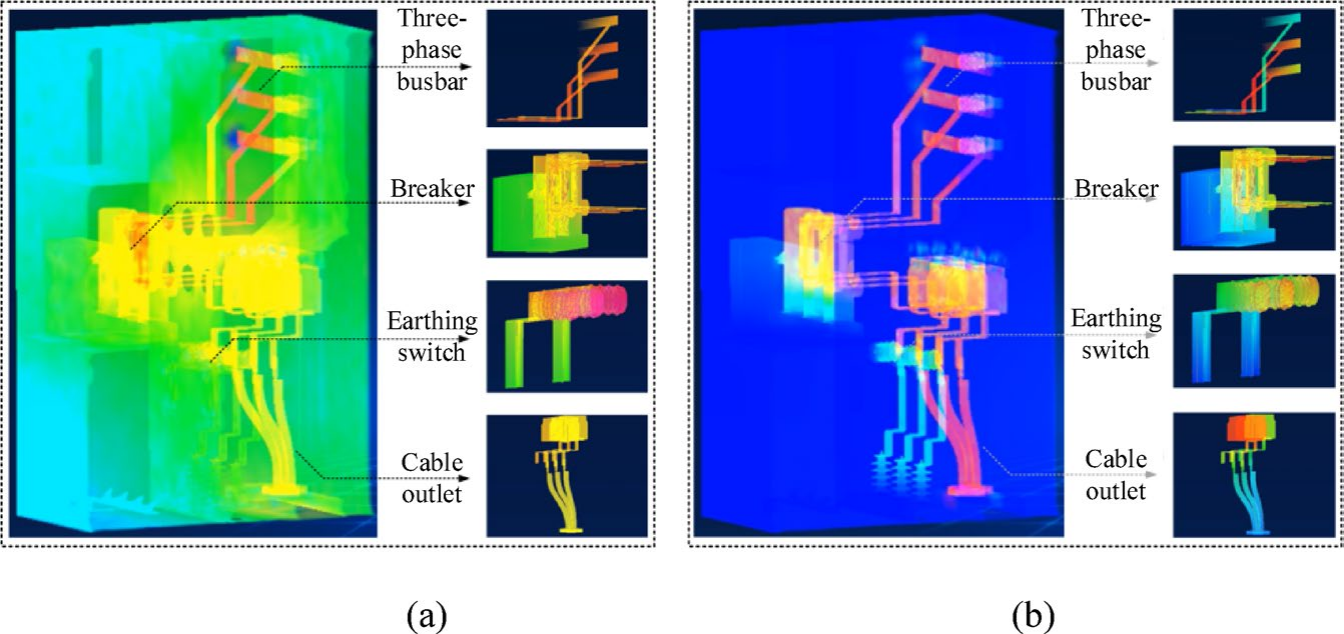
Comparison of temperature surrogate model outputs: (**a**) digital twin temperature field online simulation; (**b**) digital twin electric field online simulation.

For the online simulation of the switchgear DT, dimensionality reduction and deduplication were performed on the node data before real-time transmission. The original 449,389 simulation nodes were reduced to 181,926 and further deduplicated to 29,400 nodes, greatly improving data transmission efficiency. These 29,400 unique nodes were transmitted to Unity, where the complete set of triangles and vertices was reconstructed based on their indices, enabling the switchgear model reconstruction and point cloud rendering. The real-time rendering of the 3D point cloud for temperature and electric field data from the surrogate model is shown in Fig. 10. Figure 10(a) presents the cloud map reconstruction of the switchgear’s overall temperature output, and Fig. 10(b) shows the electric field cloud map with adjusted hue attributes in the HSV model.

#### (1) thermal field verification

**Fig. 11:**
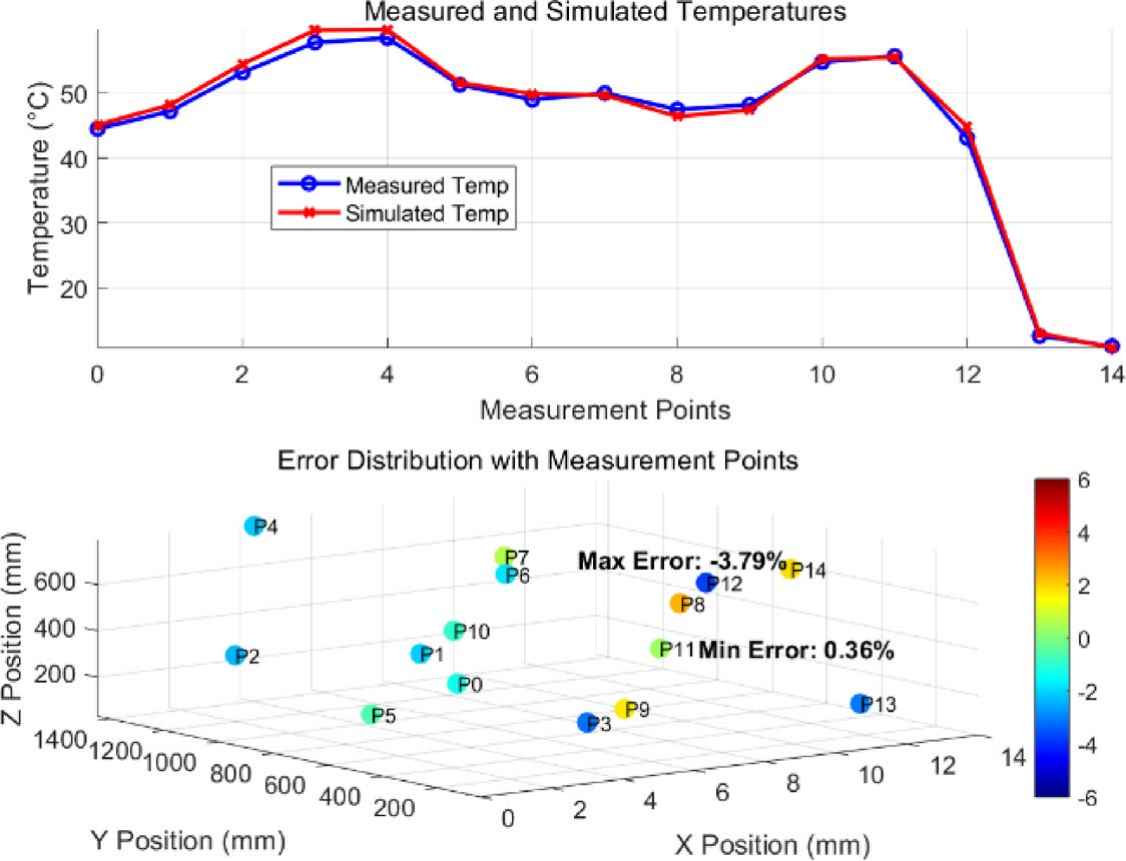
Comparison of temperature data from busbar and circuit breaker test configurations.

To verify the effectiveness of the finite element simulation model, an experimental platform was established consisting of a single-phase induction regulator, a power switchgear, a high-precision current transformer, and a thermocouple monitoring device to conduct temperature testing and obtain temperature rise data. Fifteen monitoring points, numbered 0 to 14, were placed inside the switchgear. Points 0 to 12 were distributed along the busbar inlet, the connection points, the dynamic and static contacts of the circuit breakers inlet, and the bolts connecting various components of the circuit breaker. Three-phase data were collected from these points. Points 13 and 14 correspond to the inaccessible surface inside the cabinet and the accessible surface of the cabinet, respectively. The measured and simulated temperature data are compared in Fig. 11. The comparison shows minimal error between the two sets of data, with the maximum error being − 3.79%, validating the reliability of the thermal field simulation model.

#### (2) electric field verification

In type tests, electric field qualification is primarily assessed based on apparent discharge quantity. To verify the reliability of the electric field simulation model of the switchgear under the field strength control requirement at 42 kV, a comprehensive simulation was conducted. The permissible electric field strengths are 5.25 kV/mm for high-voltage conductors in air and 2.98 kV/mm for epoxy resin surfaces. The simulation was based on a complete switchgear model, retaining the vacuum interrupter structure of the circuit breaker while isolating it from the enclosure. The busbar at the grounding switch was designated as the output terminal. Key components such as the circuit breaker, post insulators, contact box, and busbar were identified as high field intensity regions, with a 42 kV excitation applied. Simulation results indicate that the maximum field strength on conductor surfaces is 3.2 kV/mm, located at the junction between the outgoing busbar and the current transformer. The maximum field strength on insulation surfaces is 1.7 kV/mm, observed at the bend of the contact box surface. All values are below the permissible thresholds specified in national standards, confirming the reliability of the constructed electric field simulation model.

### Discussion: multisource feature data fusion and diagnostic analysis

**Fig. 12:**
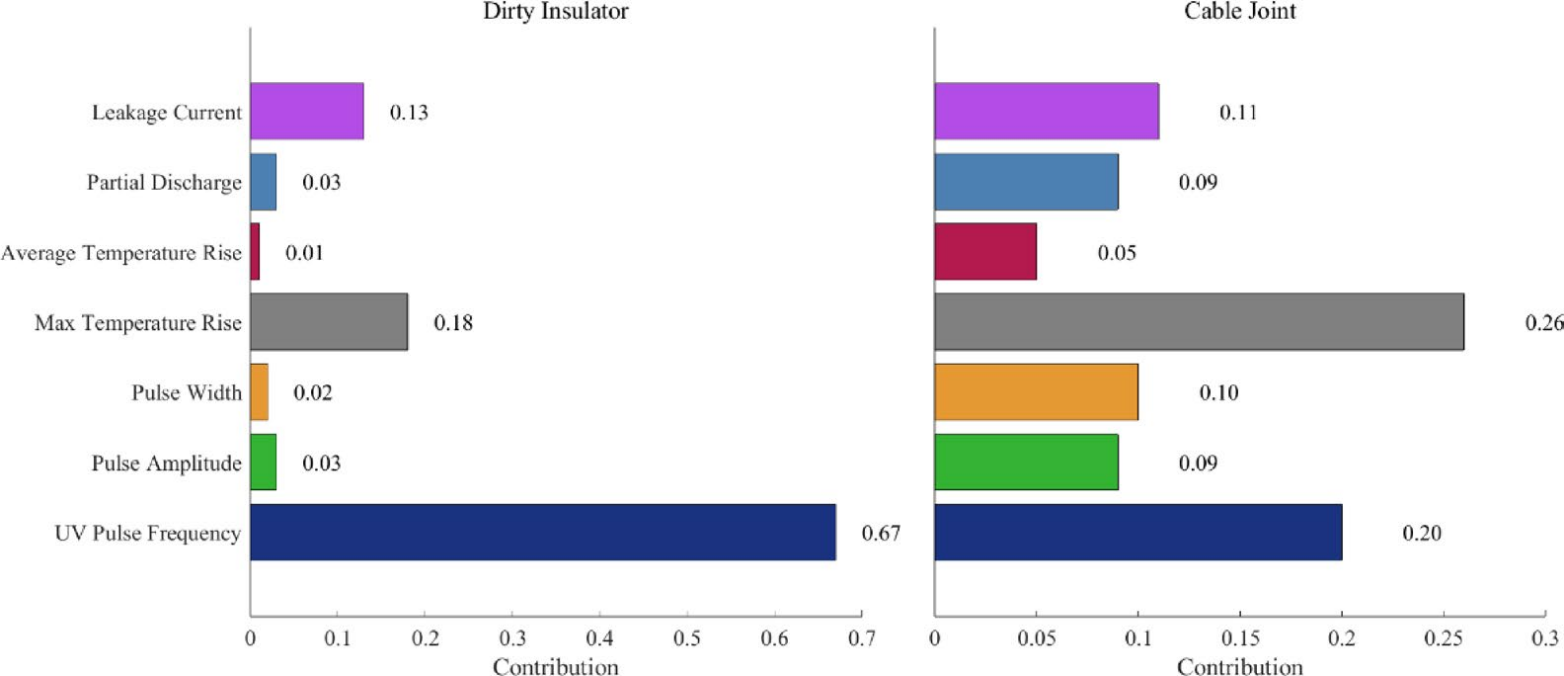
Feature importance evaluation of typical failures.

The parameters contribution for insulators and cable joints is shown in Fig. 12.

Under polluted conditions, ultraviolet (UV) pulse frequency strongly correlates with partial discharge (PD) quantity. However, PD data and UV pulse parameters exhibit significant fluctuations at high pulse counts, reducing reliability. Experiments reveal that leakage current trends align with typical insulator failure, which also causes abnormal temperature rise. Despite material and structural differences, UV pulse frequency and infrared temperature show similar trends during insulation failure. As leakage current cannot be monitored in real-time, UV pulse frequency and maximum temperature rise are selected as key indicators of switchgear failure.

Using the feature extraction algorithms, UV pulse frequency, width, and infrared temperature rise were processed. Through statistical analysis, the faults were categorized into three levels as shown in Table 2: Level I - Normal operation, with slight corona or glow discharge; Level II - Spark discharge, indicating some insulation performance but requiring timely maintenance; and Level III - Flashover or through discharge, where insulation is damaged and requires emergency repair or replacement.

**Table 2:** Equipment operation state classification.

Discharge Level	Pulse Amplitude (V)	Pulse Width (ms)	Temperature Rise (°C)	State Index	Description
I	4 ~ 10	0.76 ~ 0.88	0 ~ 2.0	0.85 ~ 1	Corona Stage
II	2 ~ 5	0.68 ~ 0.84	2.0 ~ 6.3	0.65 ~ 0.85	Spark Stage
III	1 ~ 3	0.68 ~ 0.80	≥ 6.5	0 ~ 0.65	Breakdown Stage

Before validating the diagnostic model, to detect cross-layer errors in the ANFIS, we generated noisy input data using Monte Carlo simulation to simulate measurement noise or disturbances at the input layer, assuming a ± 5% range. The experiment was repeated 1000 times, and the distribution of data errors from the input to the output layer was statistically analyzed, as shown in the Table 3.

**Table 3:** Error propagation analysis across ANFIS layers.

Layer	Input Error Range	Output Error Range	Amplification Factor
Membership	± 5%	± 4.2%	0.84
Rule	± 4.2%	± 3.8%	0.90
Decision	± 3.8%	± 3.5%	0.92
Final Output	± 3.5%	± 3.3%	0.94

This experiment demonstrates that the error decays during propagation (with amplification factors all < 1). The final output error is 34% lower than the input error (± 5% to ± 3.3%). The ANFIS architecture does not exhibit the “layer-by-layer amplification” effect. Furthermore, the low amplification factor of 0.92 at the decision layer demonstrates that the fuzzy rule base effectively suppresses disturbances. This decay phenomenon, combined with the hybrid learning algorithm of least squares and gradient descent, provides a closed-loop validation, confirming the loss function’s ability to block error propagation paths.

We extract 2,700 sets of PD pulses and maximum temperature rise data from the DT model, considering different voltage levels and physical conditions of high-voltage switchgear under abnormal states and faults of polluted insulators and cable joints. Based on the state level classification and the correlation between the state feature data and the severity of insulator faults, an empirical state index is set. From this, 2,000 sets of data are randomly selected as the training set for the fault diagnostic system network, with 150 sets from Level I, 150 sets from Level II, and 400 sets from Level III used as the test set.

**Fig. 13:**
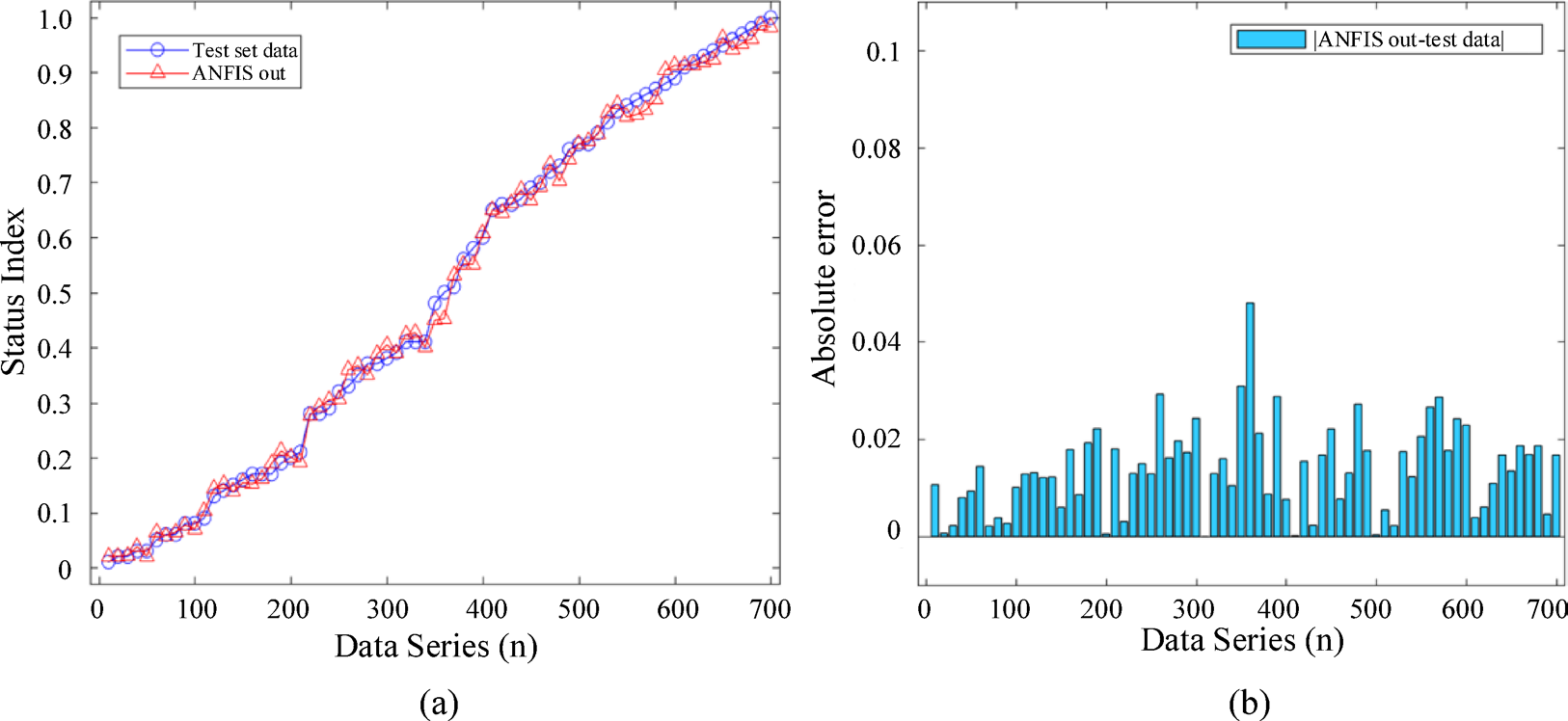
Fault diagnosis network verification for test set data: (**a**) comparison between network output and state index; (**b**) network output and absolute error of the test set.

After sorting the 700 test set data by ascending state index, the input to the ANFIS fault diagnosis network is shown in Fig. 13. In Fig. 13(a), the red triangle markers represent the ANFIS output for each test data group, which closely aligns with the blue circle markers, indicating a high degree of overlap in values and confirming the high fit of the fault diagnosis network. The network output and state index are strongly correlated, showing effective interpretation of the input state features. In Fig. 13(b), this correlation is quantified numerically, showing that the absolute error of the ANFIS output is less than 0.06 for each test data group, demonstrating the high accuracy of the fault diagnosis system.

**Fig. 14:**
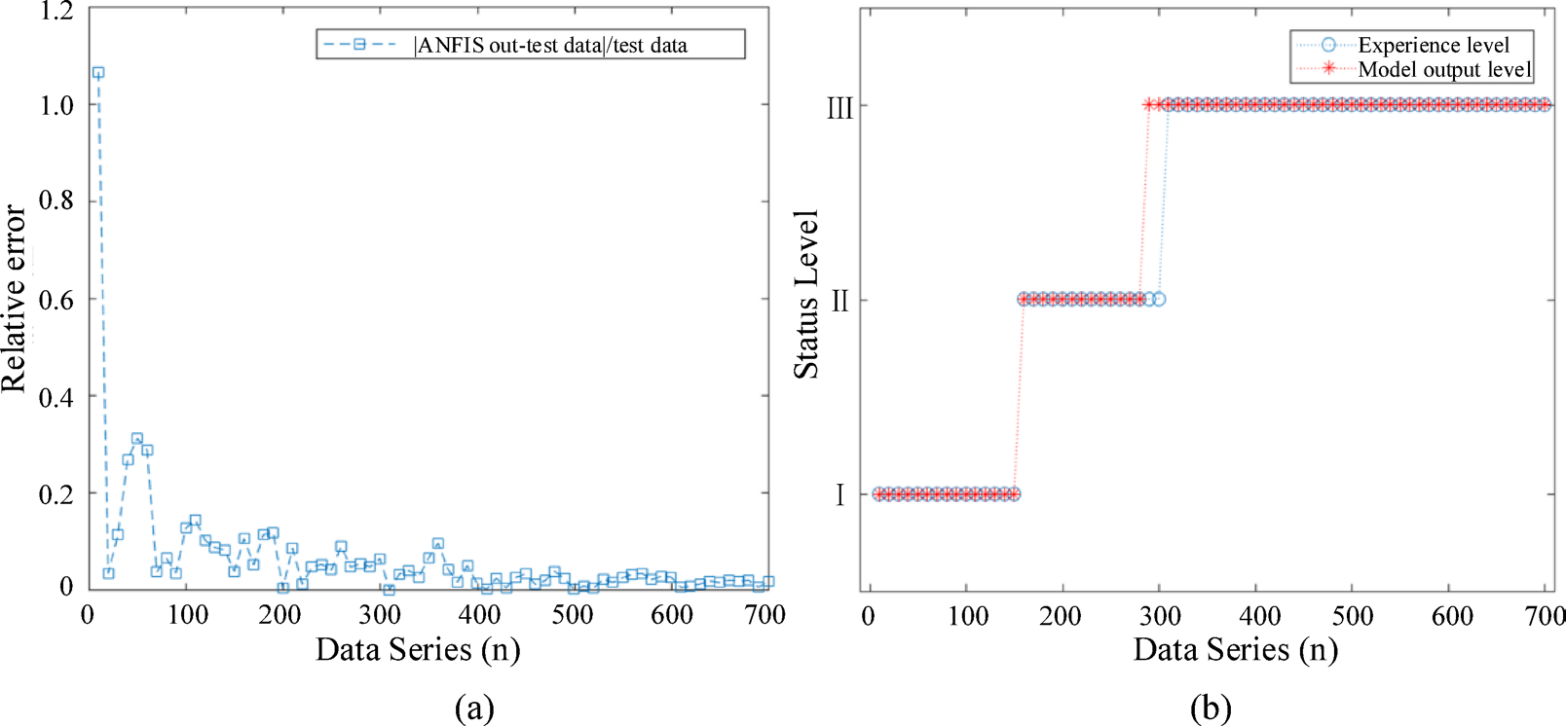
State-level outputs of the fault diagnostic model: (**a**) Relative error of network outputs (**b**) Evaluation of state-level outputs.

Furthermore, to evaluate the deviation of calculations under different state indices, Fig. 14(a) quantifies the relative error for each data group. As seen, for data with lower initial state indices, the relative error can reach up to 106% when using only the output state index. Therefore, combining the state index evaluation with the analysis in Fig. 14(b), which assesses the state level, improves the fault tolerance and reduces the impact of low state index values.

The misclassification points occur at the boundary between Level II and Level III, with empirical indices of 0.65 and 0.66, and ANFIS output indices of 0.6498 and 0.6445. However, considering the combined evaluation of the state index, the output still provides a reasonable explanation of the switchgear’s operational status. The overall accuracy for the test set reaches 93.4%, proving the reliability of the fault diagnosis model that combines state indices and levels.

In the comparison of key performance indicators among various fault diagnosis models, as shown in Table 4, the proposed ANFIS approach based on the T-S fuzzy model demonstrates overall superior performance. It achieves the highest accuracy (93.4%), precision (93.2%), recall (92.1%), and F1-score (92.6%), reflecting its strong diagnostic capability. Despite not being the fastest model in terms of computation time (8.7 s, slightly longer than SVM and DT), it maintains a good balance between performance and efficiency. Moreover, it shows the highest noise tolerance robustness, with only a 2.3% accuracy reduction under 30% noise interference, and the lowest cross-validation accuracy variance (2.1%), indicating strong robustness and stable generalization across different data subsets. These advantages make it a highly competitive and reliable model for fault diagnosis of high-voltage switchgear in practical industrial scenarios.

**Table 4:** Comparison of key performance indicators for different fault diagnosis models.

Method	Accuracy (%) ↑	Precision (%) ↑	Recall (%) ↑	F1-Score (%) ↑	Computation Time (s) ↓	Noise Tolerance Robustness (%) ↑	Cross-validation Accuracy Variance (%) ↓
BPNN	85.2	83.5	81.7	82.6	15.4	−7.4	3.7
SVM	88.1	87.5	85.4	86.6	10.2	−5.5	2.9
DT	82.5	80.9	79.2	80.0	9.8	−10.5	4.2
Fuzzy	83.7	82.1	80.8	81.4	**7.3**	−12.1	4.7
**This research**	**93.4**	**93.2**	**92.1**	**92.6**	8.7	**−2.3**	**2.1**

### Summary and conclusions

This study develops a DT-based online fault diagnosis model for high-voltage switchgear, realizing real-time sensor information monitoring, 3D field simulation, and intelligent fault diagnosis, thus providing intuitive guidance for operational maintenance. The key findings are summarized as follows:

First, the constructed 3D model of the KYN28-12(Z) switchgear, coupled with a thermal-electric field FEA model, accurately identifies internal risk zones through experimental validation, with a temperature field simulation error of less than 8% compared to test data, confirming its reliability for DT modeling. Second, the reduced-order surrogate model, integrating mesh coarsening, dictionary tree deduplication, and KNN algorithms, achieves a 90% reduction in nodes while maintaining field extreme value errors below 5%. Combined with radial basis function (RBF) interpolation, the model ensures trustworthiness and continuity above 0.91, with a response time in seconds, supported by TCP-based local loopback communication for real-time 3D field visualization. Third, the ANFIS-OCT hybrid framework, using UV pulse frequency and maximum temperature rise as key features, achieves a fault recognition rate of 93.4% on the test set, with only a 2.3% accuracy drop under 30% noise interference. This demonstrates its robustness in complex operating environments.

Overall, the proposed DT-based diagnosis model realizes accurate virtual-real mapping of switchgear states, providing a reliable technical solution for intelligent operation and maintenance of power systems. Its high precision and strong anti-interference capability validate its potential for practical engineering applications. Future work will focus on optimizing sensor deployment and conducting economic analysis for digital twin implementation in 10 kV switchgears. Additionally, dynamic response performance under transient conditions will be evaluated to enhance practical applicability.

## Data Availability

The datasets used and/or analysed during the current study available from the corresponding author on reasonable request.
